# The Neural Gut–Brain Axis of Pathological Protein Aggregation in Parkinson’s Disease and Its Counterpart in Peroral Prion Infections

**DOI:** 10.3390/v13071394

**Published:** 2021-07-18

**Authors:** Michael Beekes

**Affiliations:** Prion and Prionoid Research Unit, ZBS 6-Proteomics and Spectroscopy, ZBS-Centre for Biological Threats and Special Pathogens, Robert Koch Institute, Nordufer 20, 13353 Berlin, Germany; beekesm@rki.de; Tel.: +49-30-18754-2396

**Keywords:** Parkinson’s disease, peroral prion infections, alpha-synuclein, prion protein, seeds, spread, enteric nervous system, vagus nerve

## Abstract

A neuropathological hallmark of Parkinson’s disease (PD) is the cerebral deposition of abnormally aggregated α-synuclein (αSyn). PD-associated αSyn (αSyn^PD^) aggregates are assumed to act, in a prion-like manner, as proteinaceous nuclei (“seeds”) capable of self-templated propagation. Braak and colleagues put forward the idea of a neural gut-brain axis mediating the centripetal spread of αSyn^PD^ pathology from the enteric nervous system (ENS) to the brain in PD. This has sparked great interest and initiated passionate discussions both in support of and opposing the suggested hypothesis. A precedent for the spread of protein seeds or seeding from the gastro-intestinal (GI) tract to the central nervous system (CNS) had been previously revealed for pathological prion protein in peroral prion infections. This article scrutinizes the similarities and dissimilarities between the pathophysiological spread of disease-associated protein aggregation along the neural gut–brain axis in peroral prion infections and PD. On this basis, evidence supporting the proposed neural gut–brain axis in PD is concluded to be not as robust as that established for peroral prion infections. New tools for the ultrasensitive detection of αSyn^PD^-associated seeding activity in archived or fresh human tissue samples such as real-time quaking induced conversion (RT-QuIC) or protein misfolding cyclic amplification (PMCA) assays can possibly help to address this deficit in the future.

## 1. Introduction

**Figure 1:**
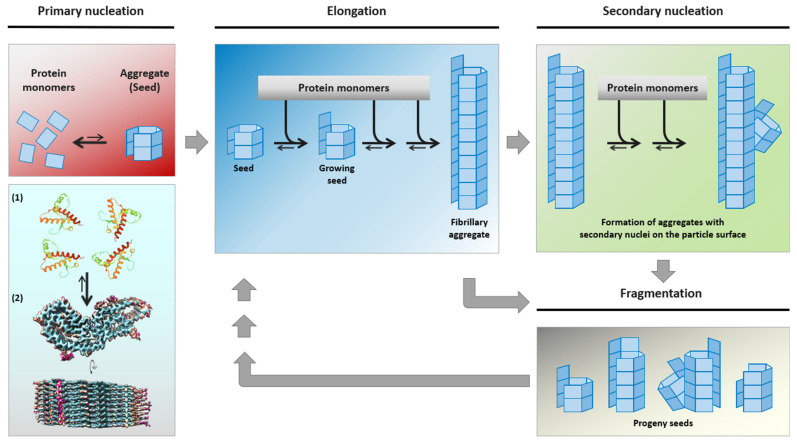
Mechanistic model of nucleation-dependent protein aggregation thought to underly the self-assembly and propagation of pathological αSyn^PD^, PrP^TSE^ or other proteinaceous seeds in PD, TSE and further neurodegenerative protein aggregation diseases, respectively. Under certain conditions, (usually monomeric) protein conformers can spontaneously, or driven by genetic factors, assemble into β-sheet-rich aggregates that constitute self-replicative protein particles, or seeds. Such initial seed formation, referred to as primary nucleation, is controlled by a high kinetic barrier. Primary nucleation is exemplarily depicted for hamster PrP, schematically showing the aggregation of PrP monomers (1, [7]) into a PrP^TSE^ seed (2, [8]). Once proteinaceous seeds have been endogenously formed, or exogenously entered the organism, they can swiftly recruit and attach further monomers of their constitutive proteins. In this process of elongation, new aggregate mass is generated by the attachment of monomeric species to the ends of the seeding-active particles. Additionally, secondary nucleation may occur by the formation of new nucleation sites on the particle surface. When protein particles with primary or secondary nucleation sites fragment into smaller aggregates, progeny seeds enter the replication cycle and further propagate the pathological protein state. The figure was produced, with modifications, following a previously published template [4] in consideration of Meisl et al. [9]. Credits for mounted image components: (1) https://www.rcsb.org/structure/4YXL (accessed on 16 July 2021): crystal structure of Syrian hamster prion protein complexed with POM1 FAB is licensed under the PDB Privacy and Usage Policy, and (2) https://www.biorxiv.org/content/10.1101/2021.02.14.431014v2 (accessed on 16 July 2021): structure of an infectious mammalian prion is licensed under the Creative Commons CC0 1.0 Universal (CC0 1.0) Public Domain Dedication.

The deposition of misfolded and aggregated alpha-synuclein (αSyn) in cerebral neuronal cell bodies or processes (referred to as Lewy bodies [LB] or Lewy neurites [LN], respectively [1]) and associated with synapses [2] is a neuropathological hallmark of idiopathic Parkinson’s disease (abbreviated in the following simply as PD, since other forms of Parkinson’s disease will not be addressed in this review). Pathological αSyn species of PD (αSyn^PD^) are assumed to act as proteinaceous nuclei (“seeds”) which recruit endogenous cellular or post-translationally modified αSyn and integrate it into their own misfolded oligomeric or polymeric aggregate structure. The seeding activity of αSyn^PD^ is thought to mediate, in a process of nucleation-dependent aggregation [3], self-templated propagation of the pathological protein state [4,5,6] (Figure 1).

Pathological accumulation of αSyn^PD^ in the brain is accompanied by a progressive loss of dopaminergic neurons in the substantia nigra pars compacta, causing a reduction of the neurotransmitter dopamine and thereby affecting neurological feedback loops in the basal ganglia. This results in the manifestation of PD-typical motor deficits such as tremor, bradykinesia, rigor and postural instability [10]. 

However, αSyn^PD^ aggregates do not exclusively occur in the central nervous system (CNS) but have been detected, too, in the enteric nervous system (ENS) of the gastro-intestinal (GI) tract of PD patients. Concomitantly with this, PD is associated with non-motor symptoms that often manifest prodromally in the GI tract, e.g., in the form of constipation or reduced peristalsis [11]. This gave rise to hypothesize both a link between clinical PD manifestations in the GI tract and brain, and αSyn^PD^ pathologies in these tissues as a cause for these disease symptoms [12,13]. The idea of a gut–brain axis in PD was put forward by Braak and colleagues who in 2003 had presented a staging system for PD according to identified patterns of αSyn^PD^ deposition in autopsy cases [14]. Based on this staging system they proposed that a pathogen, first considered as a neurotropic virus and later increasingly emphasized as misfolded and aggregated αSyn with prion-like properties, propagates from initial foci in the ENS retrogradely via efferent fibres of the vagus nerve to the dorsal motor nucleus of the vagus nerve (DMV, elsewhere also abbreviated as DMNV) in the brain stem, and from there further on to other cerebral target sites including the substantia nigra [15]. Together with a second proposed spreading pathway for αSyn^PD^ pathology, from the nasal cavity via the olfactory bulb into the temporal lobe, the concept became known as the dual-hit hypothesis of PD [16,17]. 

A precedent for a vagal gut–brain axis mediating brain invasion by proteinaceous seeds or seeding from the GI tract had been revealed for peroral prion infections several years before Braak and colleagues presented their concept. Prions are infectious self-replicating protein seeds that are thought to essentially consist of pathologically misfolded and aggregated conformational isomers of the cellular prion protein (PrP^C^), referred to as PrP^Sc^ or PrP^TSE^ [18,19]. They are the causative agents of transmissible spongiform encephalopathies (TSE), such as scrapie, bovine spongiform encephalopathy (BSE), chronic wasting disease (CWD) or Creutzfeldt–Jakob disease (CJD) and its BSE-derived variant (vCJD) [20]. 

The putative spread of aSyn^PD^ pathology along the gut–brain axis and within the brain in PD, as well as the potentially prion-like properties of αSyn^PD^ seeds and their disputed role as a driver of clinical disease, have been summarized and discussed in numerous scientific articles and reviews over the past years [21,22,23,24,25,26,27,28]. However, only rarely and then mostly summarily has reference been made to the available original data and reports on the actual spread of prions along the gut–brain axis in peroral prion infections. This can be traced back even to the roots of the concept of a gut–brain axis in PD. Already, Braak and colleagues only indirectly and rudimentarily quoted the original findings on the propagation of prions along the gut–brain axis in peroral TSE infections that provided an obvious blueprint for their spreading model, particularly with regard to the retrograde axonal routing along the vagus nerve from the ENS to the DMV [15]. 

Such omission does not seem helpful for an optimal comparative evaluation of the available data on the spread of PrP^TSE^ and αSyn^PD^ pathologies along the gut–brain axis in peroral prion infections and PD, respectively. In one of the few papers that include original literature on the gut–brain axis in peroral prion infections in somewhat more detail, the authors state “An important research goal is to elucidate the degree of similarity between pathophysiological processes in PD and those of true prion disease” [29]. In line with this approach, this article intends to provide a review of neural pathways along the gut–brain axis thought to mediate centripetal spread of PrP^TSE^ and αSyn^PD^ seeds or seeding from the alimentary tract to the CNS. Key findings obtained in this regard for authentic mammalian prions and PrP^TSE^ will be summarized and compared with available data on PD-associated αSyn forms. On this basis, similarities, dissimilarities, outstanding questions and possible future research directions will be highlighted. This review does not include findings on pathological αSyn species from non-PD αSyn aggregation diseases (e.g., dementia with Lewy bodies [DLB] or multiple system atrophy [MSA]), or on in vitro generated fibrillar or non-fibrillar αSyn assemblies, because such αSyn species may not necessarily share the pathophysiological properties of αSyn^PD^ with respect to the hypothetized gut–brain axis in PD.

## 2. The Established Neural Gut–Brain Axis in Peroral Prion Infections

Early evidence for a centripetal spread of prion infection from the GI tract to the spinal cord via components of the enteric and autonomic nervous system was obtained in infectivity studies with mice that had been intragastrically challenged with scrapie [30]. The dynamics of the spread of perorally acquired prion infections from the GI tract to the spinal cord and brain were subsequently further elucidated in hamsters perorally exposed to the 263 K scrapie agent [31,32,33,34,35,36]. These studies used bioassays in animals for the detection of prion infectivity, as well as different techniques for the visualization of PrP^TSE^, which was established in many studies as a reliable biochemical marker for TSE infectivity [31,33,37,38,39,40,41], to map the temporal–spatial spread of infection after a peroral challenge with prions. Throughout this review, the abbreviation PrP^TSE^ will be used to collectively designate pathological forms of PrP associated with prion infection that can be detected in affected animals and humans by analytical methods such as immunohistochemistry (IHC) [32,42], Western blotting [31,43] or paraffin-embedded tissue blotting [44]. Western and paraffin-embedded tissue blotting detect partially proteinase K (PK)-resistant forms of PrP^TSE^, while IHC visualizes aggregated deposits of this protein.

Studies using the hamster model of peroral 263 K scrapie infection revealed, among other findings, that initial foci of PrP^TSE^ deposition in the nervous system—and thus neuroinvasion—occurred in submucosal and myenteric ENS ganglia of the small intestine [32,34]. The small intestine (as with other parts of the gut and the stomach) is innervated, partly via ENS ganglia, by the vagus and splanchnic nerves, efferent fibres of which belong to the parasympathetic and sympathetic nervous system, respectively. This constitutes a dual neural gut–brain axis, along which the infection was conveyed directly or via the spinal cord, respectively, to the brain [32,33,35,36]. Low levels of prion infectivity could be directly detected in the cervical vagus nerve of clinically diseased hamsters perorally challenged with 263 K scrapie [32]. That the vagus nerve can harbour PrP^TSE^ had already previously been found in hamsters intraperitoneally challenged with scrapie [45]. While these findings indicated the vagus nerve as a conduit for prion infectivity or PrP^TSE^, taken in themselves, they did not provide information on the direction of spread.

However, as reviewed in detail elsewhere [46], the centripetal spread of perorally acquired 263 K scrapie infection in hamsters was progressively deciphered [31,32,33,34,35,36], and revealed to ascend retrogradely via efferent fibres of the vagus and splanchnic nerves innervating the gut, to the DMV and commissural nucleus of the solitary tract in the medualla oblongata of the brain, and to the celiac/mesenteric ganglion complex and intermediolateral cell column (IML) in the thoracic spinal cord, respectively. In this context, it is noteworthy that the time window for the detection of PrP^TSE^ pathology in the ENS only was obviously so narrow that it could not be hit in the hamster model. The ENS and DMV of hamsters that had been perorally challenged with identical doses of 263 K scrapie prions were still found to be immunohistochemically negative for PrP^TSE^ at 45 and 56 days post infection (dpi), respectively, while both sites became practically simultaneously positive for PrP^TSE^ at 62/63 in two out of four (2/4) animals and at 60 dpi in 2/6 animals [32,47]. From the sites of cerebral and spinal neuroinvasion, the infection propagated, apparently along defined neuroanatomical projections and in a specific temporal–spatial sequence, within the brain and spinal cord [35]. In the latter, infection showed a bidirectional spread that included ascension to the brain. Centrifugal routing from the CNS appeared to be responsible for subsequent infection of sensory nodose and dorsal root ganglia of the vagus and splanchnic nerve circuitries, respectively, although a direct spread from the viscera along sensory fibres to the nodose and dorsal root ganglia could not be ruled out [32]. Detailed pictorial representations summarizing these findings on the spread of infection along the gut–CNS axis in hamsters perorally infected with scrapie have been provided by McBride et al. [32] or elsewhere [48]. 

Mostly identical or at least largely similar findings have been reported regarding the centripetal spread of prions from ENS ganglia to the brain and spinal cord via the vagus nerve circuitry (DMV of the vagus nerve–commissural nucleus of the solitary tract–nodose ganglia) and splanchnic nerve circuitry (celiac and mesenteric ganglion complex–IML–dorsal root ganglia), respectively, in perorally/alimentarily acquired scrapie of sheep [49,50,51], CWD of cervids [52,53], and BSE of cattle [54].

In light of additionally reported findings, some further refinements were made to the dual gut–brain spreading model of peroral prion infections. These include the identification of the sympathetic ganglia chain in cattle perorally challenged with BSE as an additional spreading pathway bypassing the spinal cord and mediating infection of the obex and DMV beside the vagus nerve [55]. In this study, it became evident again that, if at all, only a very narrow time window existed in which PrP^TSE^ pathology was already detectable in the ENS but not yet in the brain. Of eight animals whose ENS and brainstem were examined between 16 and 28 months post infection, only one was found to be affected in the ENS without detectable PrP^TSE^ or infectivity in the obex or caudal medulla, respectively.

After foodborne infection of non-human primates (cynomolgus monkeys) with BSE prions, early infection of ENS ganglia was observed, but indications for brain invasion via the vagus nerve as typically seen in peroral prion infections of rodents and ruminants were found in only one monkey. In contrast, most macaques showed invasion of the CNS primarily at the level of the lumbar spinal cord, which was obviously mediated by anterograde spread along visceral afferents. PrP^TSE^ accumulation in dorsal root ganglia preceded PrP^TSE^ deposition in the corresponding dorsal horn, with initial accumulations in the substantia gelatinosa [56]. 

In vCJD patients, i.e., humans that acquired the infection presumably due to an alimentary exposure to BSE prions, PrP^TSE^ was found in sympathetic celiac, superior mesenteric and stellate ganglia [57], as well as in dorsal root ganglia [58]. This supported a role for the sympathetic nervous system in prion propagation from the gut to the CNS. In one study on the neuropathology of vCJD, ENS ganglia and parasympathetic structures also showed positive imunohistochemical staining, but the author stressed that these findings need to be interpreted with caution [58]. 

Additionally to the described centripetal and centrifugal spreading pathways of the autonomic and peripheral sensory nervous system, the neural routing of TSE agents in peroral prion infections includes further aspects such as centrifugal spread from the CNS to muscles [59,60] and skin [61]. Furthermore, peroral TSE infections may also result in a blood-borne dissemination of prions from the periphery to the brain, as will be outlined in the following paragraph. 

## 3. Haematogenous Neuroinvasion of the Brain in Peroral Prion Infections

Comprehensive studies in scrapie- or BSE-infected sheep that included peroral infections have shown that the haematogenous route can represent a parallel or alternative pathway to cerebral neuroinvasion from the ENS via the autonomic nervous system [62]. Access of prions to the bloodstream after ingestion of TSE agents is thought to occur following invasion of and replication in components of the gut-associated lymphoid tissue (GALT), possibly among other pathways, via infected free-ranging lymphoid cells that cross to the efferent lymph in the cortical and paracortical sinuses of GALT-draining lymph nodes [46]. 

In cases of haematogenous prion spread, initial PrP^TSE^ accumulation also consistently occurred in the DMV and its related circumventricular organ with a diminished blood–brain barrier (i.e., the area postrema) [63]. Therefore, special attention had to be paid to the presence or absence of pathological protein aggregates in circumventricular organs when interpreting findings of PrP^TSE^ pathologies in the DMV in tracing studies. Experiments in hamsters on the neural gut–brain axis in peroral 263 K scrapie infections did not reveal any evidence for haematogenous spread of infection to the brain. PrP^TSE^ was not detected early in infection at sites with an impaired blood–brain barrier, such as the area postrema [35] or the choroid plexus [32]. In addition, routing via the blood would not be consistent with the observed temporal–spatial pattern of PrP^TSE^ targeting in the autonomic nervous system, spinal cord and brain [32]. 

## 4. The Postulated Neural Gut–Brain Axis in Parkinson’s Disease (PD)

Starting in 2003, Braak and colleagues reported a pathological staging system for PD, which was based on an identified temporal–spatial pattern of LB and LN pathology that could be apparently well aligned with the course of the disease [14,64]. The authors reported that cerebral Lewy pathology (LP) in PD initially occurs in the olfactory bulb and the DMV before propagating to other cerebral areas including the substantia nigra [14,15,64]. Their findings led Braak and colleagues to propose the “dual-hit” hypothesis postulating that sporadic PD starts at two sites in the body, i.e., neurons of the nasal cavity and neurons of the ENS in the gut or stomach, due to exposure to an unknown neurotophic pathogen triggering LP [15,16,17]. From these sites, the LP stimulating agent was hypothesized to invade the brain in a specific temporal–spatial pattern, i.e., anterogradely along the olfactory tract to the temporal lobe and retrogradely along the vagus nerve to the DMV, respectively.

Braak and colleagues reasoned that the hypothesized neurotropic pathogen could possibly induce conformational changes in normal αSyn molecules provoking their aggregation without becoming itself aggregated and integrated into the pathological αSyn^PD^ deposits, or that the pathogen could possess unconventional prion-like properties and might consist of misfolded αSyn fragments [15]. The implication of a prion-like self-templated propagation of the pathological protein states in PD, together with the additional finding that pathological αSyn^PD^ aggregates can spread from neuron to neuron [65,66,67,68] delivered a plausible mechanistic basis for the proposed stereotypic temporal–spatial spread of αSyn pathology in PD. 

Once self-propagating αSyn^PD^ aggregates, or seeds, have been formed in such a scenario in olfactory and enteric neurons, αSyn^PD^ pathology further propagates in the nervous system by the spread of αSyn seeding (that may or may not include a translocation of αSyn seeds) in highly defined temporal–spatial patterns, apparently trans-synaptically, along established neuronal projections and circuits to the initial target areas in the brain. It has been demonstrated in animal experiments that vagal axons and terminals from preganglionic efferent neurons located in the DMV show immunoreactivity for αSyn, and that the latter neurons have synaptic contacts with αSyn-positive neurons in the myenteric plexus of both the stomach and duodenum [69]. This supports the vagus nerve as an αSyn-expressing parasympathetic pathway for the retrograde propagation of αSyn pathology from the ENS to the CNS.

**Figure 2:**
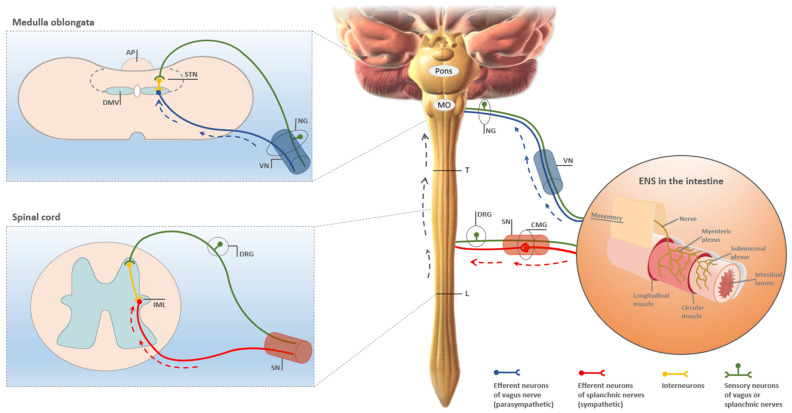
Pictorial representation of the neural gut–brain axis thought to be involved in the centripetal spread of pathological protein aggregation from the intestine to the CNS in both peroral prion infections as well as PD. As established in great detail for perorally acquired TSE early spread of prions and PrP^TSE^ pathology to the CNS occurs from ENS ganglia in a retrograde direction along parasympathetic and sympathetic efferents of the vagus and splanchnic nerves, to the DMV in the medulla oblongata and the IML in the spinal cord, respectively (reviewed in: [46,48]). A similar concept was subsequently proposed for the propagation of LP in PD from enteric ganglia in the gastrointestinal tract to the CNS, initially with respect to parasympathetic and later also with respect to sympathetic spreading pathways [14,73,76]. Dashed blue and red arrows indicate retrograde spread via efferent parasympathetic or sympathetic projections of the vagus or splanchnic nerves, respectively, for both peroral prion infections and PD. Dashed grey arrows mark spinal ascension of PrP^TSE^ pathology or LP to the brain following invasion of the IML at thoracic spinal cord levels of splanchnic innervation [46,73]. The figure was produced, with modifications, following templates from McBride et al. [32] and Mabbott and MacPherson [48]. Credits for mounted image components: “Blausen 0838 Sympathetic Innervation” and “Blausen 0703 Parasympathetic Innervation” by Bruce Blaus are licensed under the Creative Commons Attribution 3.0 Unported License (CC BY 3.0); “Anatomic structure of enteric plexus” (image ID FK8KPF) is licensed by Alamy Limited (Abingdon, UK, invoice number IY01945617). AP, area postrema; CMG, celiac or mesenteric ganglion; DMV, dorsal motor nucleus of the vagus nerve; DRG, dorsal root ganglion; ENS, enteric nervous system; IML, intermediolateral cell column; L, border between thoracic and lumbar spinal cord; MO, medulla oblongata; NG, nodose ganglion; SN, splanchnic nerves; STN, solitary tract nucleus; T, border between cervical and thoracic spinal cord; VN, vagus nerve.

LB and LN have also been detected in sympathetic preganglionic neurons in the IML of the spinal cord, and in postganglionic neurons of the celiac ganglion in early stages of PD [70,71,72]. Thus, an additional spreading pathway of αSyn pathology to the CNS is conceivable by retrograde axonal propagation along the splanchnic nerve to postganglionic sympathetic neurons in the prevertebral celiac ganglion. From there, invasion of the spinal cord may occur via further retrograde spread to preganglionic sympathetic neurons in the IML [73]. Klingelhoefer and Reichmann [74] concluded in a comprehensive review paper on the topic that “Current evidence indicates that PD pathology that involves alpha-synuclein propagates from the ENS by trans-synaptic cell-to-cell transmission through sympathetic and parasympathetic nerves to the DMNV and IML into the CNS”. This seems to suggest a nearly perfect cross-match with the spread of PrP^TSE^ pathology along the dual gut–brain axis in peroral prion infections. At first glance, such conclusion appears also striking when looking at pictorial representations of the neural spreading pathways of PrP^TSE^ and αSyn^PD^ pathologies suggested to operate in peroral prion infections [32,48] and PD [15,74,75], respectively (Figure 2). However, is this really how the facts are?

## 5. Is There a Neural Gut–Brain Axis in PD? Pros, Cons and Insights from Peroral Prion Infections

Before comparing the temporal–spatial spreading of PrP^TSE^ and αSyn^PD^ pathologies, i.e., of the propagation of PrP^TSE^ and αSyn^PD^ seeds or seeding, along the gut–brain axis in peroral prion infections and PD, respectively, fundamental differences with regard to the diseases must be pointed out.

First, TSE can be transmitted and are partly even contagious (e.g., ovine scrapie or cervid CWD), whereas PD is generally assumed to be non-transmissible, at least not as a full-blown syndrome. Thus, while PD-associated αSyn^PD^ seeds share nucleation-dependent protein aggregation as the molecular mechanism of their origin and propagation with PrP^TSE^ seeds, they lack the latter’s obvious clinical infectiousness. Particularly, the role of LP-associated and other forms of αSyn^PD^ as a pathogenic driver of neuronal dysfunction, death of nerve cells and symptomatic disease is still less reliably established, and more controversially discussed, than that of PrP^TSE^ for prion diseases. 

Second, when comparing the spread of PrP^TSE^ and αSyn^PD^ pathology along the gut–brain axis, a further discrepancy needs to be considered. For the mapping of spreading patterns in peroral prion infections PrP^TSE^ itself was used as a tracer. In contrast, the concept of the gut–brain axis in PD was derived less from an analysis of αSyn^PD^ pathology as a whole, but mainly based on the detection of LB and LN. However, several different studies have demonstrated that neuritic αSyn pathology is much more abundant than perikaryal inclusions in different αSyn aggregation diseases [77,78,79], and up to 90% or even more of pathological cerebral αSyn aggregates were found to not be localized in LB or LN but at presynapses in the form of much smaller micro-deposits [2,80]. This raises the fundamental question as to whether the spread of αSyn^PD^ seeds or seeding along the gut–brain axis in PD can be reliably concluded from identified patterns of LP. 

The assumption of an ascending vagal pathway conveying brain invasion of αSyn^PD^ pathology from the ENS is based on the following factors according to the essence of Braak’s concept: (i) that the DMV is an initial site of αSyn^PD^ pathology in the brain, (ii) that αSyn^PD^ pathology in the ENS should basically precede that in the DMV, and (iii) that the vagus nerve can contain and act as a conduit for αSyn^PD^. 

### 5.1. Studies Based on Human Tissues and Epidemiological Data

Braak and colleagues derived their concept from the postmortem examination of deceased humans. Therefore, they could not draw on definite longitudinal information, i.e., hard data on the evolution of LP as a function of time. This represents a noteworthy difference to the highly controlled cause-effect studies in animals that elucidated the neural gut–brain axis of PrP^TSE^ propagation in peroral prion infections. Rather, longitudinal relationships had to be inductively concluded by Braak and colleagues on the basis of plausible but partly unverified assumptions. This could provide an explanation for the fact that subsequent studies delivered differing results in that only about half of the examined PD patients were found to show patterns of LP that were consistent with Braak’s staging system [81,82,83], with some patients having been apparently free of detectable LP [84]. Attems and Jellinger reported based on the examination of 60 cases that about 18% did not follow the Braak staging scheme, and that in about 8%, the DMV was not involved despite definite aSyn inclusions in other brainstem or even cortical regions [85]. That the DMV was not displaying LP in some cases led these authors to the conclusion that it is not an obligatory transfer or trigger site in PD. Further reports in support or against Braak’s staging hypothesis for PD have been recently reviewed by Jellinger [86] with the conclusion that the validity of this staging concept warrants further studies in regard to various subtypes of PD.

Apparently more critical with respect to Braak’s hypothesis is that comprehensive postmortem studies could not confirm its interpretation of the relative prevalence, and thus the implied temporal sequence, of αSyn^PD^ pathology in the ENS and brain. Beach and colleagues [27,87] reported, that they “did not find a single case in which LB and LN were present in the peripheral autonomic networks (including the ENS) but not in the CNS” in a neuropathological survey of 417 autopsy cases. After these authors had expanded their data collection based on more than 600 whole-body autopsies, they still failed to identify a case in which the ENS or other parts of the peripheral nervous, but not the brain, exhibited LP [27].

However, as pointed out by Borghammer and Van Den Berge [88], the time window for the identification of αSyn^PD^ pathology in the ENS without involvement of the brain stem may be quite narrow and possibly comprises only a few weeks. As these authors stated, “initially highly localized gut pathology could immediately propagate to the DMV, giving rise to the first CNS pathology, and thereby disqualifying this patient from being categorized as a gut-only case”. Finding individuals with gut-only αSyn^PD^ pathology could therefore be difficult. In light of similar findings described above for peroral prion infections in hamsters and cattle, this seems to be a valid point. However, as long as the lack of human gut-only cases highlighted by Beach and colleagues [27] is not eliminated or compensated by other findings conclusively proving Braak’s postulated sequence of events, there basically remain at least three logical alternatives: (i) αSyn^PD^ pathology in PD starts simultaneously at different sites of the nervous system (e.g., in the ENS and DMV), (ii) αSyn^PD^ pathology in the ENS precedes that in the brain, or (iii) αSyn^PD^ pathology in the brain predates that in the ENS. This would also leave it uncertain as to whether αSyn^PD^ pathology spreads along the vagus nerve centripetally, centrifugally, bi-directionally or at all. 

While phosphorylated αSyn^PD^ histopathology had been detected in the vagus nerve of PD patients [89], the involvement of the vagus nerve in the development of PD was further examined in two studies that analyzed the effect of full truncal vagotomy (performed in patients for the treatment of peptic ulcer) on the risk of developing the disease. Statistical analyses using data from Danish registries indicated that full truncal vagotomy was associated with a decreased risk for subsequent development of PD, suggesting that the vagus nerve may be critically involved in PD pathogenesis [90]. Similarly, a study based on nationwide Swedish registers found suggestive evidence for a potential protective effect of truncal vagotomy against PD development [91]. However, when the Danish dataset was independently reanalyzed by a Norwegian research group with different patient categorization and methods for statistical analysis, these authors came to the conclusion “that it remains to be shown that vagotomy reduces the risk of having PD” [92]. 

Drawing definitive conclusions from these epidemiological studies is additionally complicated by a possible secondary effect of vagotomy, i.e., retrograde degeneration of neurons in the DMV [93,94,95]. Considering the observed haematogenous spread of prions to the DMV described above for specific paradigms of perorally acquired TSE, this brain region could hypothetically also provide an invasion site for αSyn^PD^ pathology not only after neural but possibly also after haematogenous spread. Although to the best of the author’s knowledge there has been no evidence reported so far that αSyn^PD^ enters the blood stream from GI tissue, such a scenario cannot be ruled out yet with absolute certainty. Vagotomy and associated retrograde degeneration of DMV neurons could then theoretically disrupt both parasympathetic and blood-borne neuroinvasion of the DMV. In this case, demonstrating a protective effect of vagotomy on subsequent development of PD would not be sufficient to prove that the vagus nerve is critically involved in the pathogenesis of the disease.

### 5.2. Studies in Animals

Perorally acquired prion infections can be accurately modelled and studied in detail in a variety of wild-type animal models, including rodents, ruminants and primates. For PD, however, neither wild-type nor transgenic animal models accurately mimicking the disease exist. While the causative neurotropic pathogens of TSE, prions, are considered as reliably established, the situation with PD is substantially different. The identity of the neurotropic pathogen in PD postulated by Braak and colleagues is unclear. If one assumes this pathogen to be composed of prion-like αSyn^PD^ particles and sets aside the open question of whether pathological forms of PD-associated αSyn are the causative drivers of the disease at all, one faces the problem of how to test Braak’s concept on the centripetal spread of LP in animal models none of which realistically replicates the disease. Several different studies addressed this challenge by trying to trace the spread of αSyn aggregates and αSyn pathology from the GI tract to the CNS. For this purpose, materials containing pathological αSyn species from patients with α-synucleinopathies or in vitro generated fibrillar or non-fibrillar αSyn assemblies, were injected into the stomach or gut wall of laboratory animals as reviewed elsewhere [26,96]. While elegantly designed and delivering important results, the actual probative value of these studies with respect to Braak’s hypothesis on the gut–brain axis in PD is questionable. 

First, non-PD αSyn species used in such tracing studies may not necessarily share the structural and biological properties of αSyn^PD^. Therefore, tracing results obtained with aggregated αSyn species other than those from PD patients cannot be straightforwardly extrapolated to the postulated gut–brain axis in PD and are therefore not included in this review. Second, tracing studies using authentic αSyn^PD^ species from PD patients have not been performed in non-invasive ways as this was carried out by simple feeding of prion-contaminated foodstuffs to study the spread of peroral TSE infections [46,48]. Rather, inoculates containing αSyn^PD^ were administered by injection into the stomach and/or intestinal wall [97,98].

One study by Holmqvist et al. [97] using invasive GI exposure of rodents to αSyn^PD^ provided strong evidence that PD-associated αSyn aggregates injected into the intestinal wall were transported retrogradely via the vagus nerve to the DMV. However, due to its very design, this study was not able to do so for a natural route of exposure such as that postulated by Braak and colleagues, and underlying peroral prion infections. Furthermore, GI injection of tissue lysates or other materials containing αSyn^PD^ may result in an artificial invasion of the blood stream and subsequent haematogenous spread to the brain with possible invasion of circumventricular organs and the DMV, as suggested by experiences from the prion field. However, control experiments effectively ruling out such haematogenous spread of their inoculums were not reported by the authors. This suggests to interpret the detection of αSyn staining in the DMV by Holmqvist et al. only cautiously as evidence for neural propagation along the vagus nerve. Irrespective of whether invasion of the DMV in the rats of this study occurred via the vagus nerve or blood stream, it should be also noted, as already suggested by Lionnet et al. [27], that there was no evidence for further propagation of αSyn pathology in this brain region. 

A second study addressing the spread of αSyn pathology after enteric injection of αSyn^PD^ containing LB extracts from patients with PD was recently reported by Arotcarena et al. [98]. These authors found in baboon monkeys indications for a bidirectional spread via the blood circulation of pathological αSyn species between the ENS and CNS. Notably, Arotcarena et al. did not find pathological αSyn lesions in the vagus nerve or DMV and concluded that these neuroanatomical sites were not involved in the observed spread of αSyn pathology in the used animal model. However, since the model animals used in this study did not show any formation of LB or LN, even after intrastriatal injection of αSyn^PD^ containing LB extracts, they do not seem to provide an ideal experimental paradigm for testing Braak’s hypothesis on the vagal spread of LP. Rather, αSyn pathology in this model was immunohistochemically characterized by pathological phosphorylation of αSyn at serine 129 (pSer129 αSyn) and increased IHC staining intensities of pSer129 αSyn species in different brain regions, with the authors concluding cautiously that “enteric injection of LB-enriched fractions might induce α-synuclein pathology throughout the nigrostriatal tract, similar to that observed after striatal injection”. 

Thus, taken together, the available data from animal studies do not support and allow to delineate gut–brain spreading scenarios of αSyn^PD^ pathology as clearly as it has been achieved for PrP^TSE^ in peroral prion infections of animals. For this reason, Braak’s hypothesis cannot be robustly validated based on the data from animal experiments reported so far. Whether this will be possible in the future is a fundamental question not only because of the lack of accurate animal PD models already mentioned above. To date, stimulation of LP by injected αSyn^PD^-containing inoculums has only been observed in one animal study (in the form of LN) [99]. Otherwise, either only the induction of diffuse pathological αSyn accumulations containing phosphorylated αSyn species [98,100] or no αSyn pathology at all [101] have been detected in the brain of recipient animals after enteric or intracerebral injection of such test materials. Furthermore, other than LB extracts purified from postmortem PD brain tissue [100], peripheral αSyn^PD^ aggregates prepared from sympathetic stellate ganglia of PD patients did not stimulate pSer129 αSyn pathology after intracerebral injection into wildtype mice [102]. While the latter findings do not necessarily challenge Braak’s hypothesis since the used mouse model did not develop LB or LN at all and the injected peripheral αSyn^PD^ preparations had not been purified from the ENS, they fit the notion that αSyn^PD^ seeds represent in several respects a much more challenging subject for neuroanatomical tracing studies than PrP^TSE^ of prion diseases.

## 6. Conclusions

### 6.1. Does the Concept of a Neural Gut–Brain Axis of Pathological Protein Aggregation Hold in PD and Which Alternative or Complementary Hypotheses Are Being Discussed?

The hypothesis of a neural gut–brain axis mediating stereotypical spread of pathological prion-like protein aggregation in Parkinson’s disease, which originated from the work of Braak and colleagues as referenced above, has undergone a remarkable development in the past few years and triggered an ongoing multifaceted and controversial discussion [27,28,29,74,96,103]. A comparison with its counterpart, the concept of a dual neural gut–brain axis in peroral prion infections, shows that the robustness of the evidence for centripetal spread of αSyn^PD^ or PrP^TSE^ pathology along the gut–brain axis in PD and peroral prion infections, respectively, is substantially stronger for the latter. As outlined, this is due in large part to the disparity in the availability of non-invasive accurately disease-mimicking animal models for detailed cause–effect studies on the spread of PrP^TSE^ and αSyn^PD^ pathology from the ENS to the brain. 

The fact that research into the neural gut–brain axis of pathological protein aggregation has faced and still faces much greater difficulties and challenges with respect to PD than it did for peroral prion infections readily explains the limitations and shortcomings pointed out above for pathophysiological PD studies conducted in this field. With the technology where it is today, and when the data and their underlying studies presented in this review are considered in their entity, this author feels it too early to judge whether the balance of evidence and plausibility is more in favour of a neural gut–brain axis of spreading αSyn^PD^ pathology in cases of PD than against it. 

That said, this conclusion requires further consideration. Braak’s staging system of LB pathology does not hold for all PD patients [26,104]. While this does not compromise the staging system per se, it challenges its general validity for PD and that of the dual-hit hypothesis postulating centripetal neural spread of LB pathology from the GI tract and nasal cavity to initial target sites in the brain. It has been suggested that in some cases of PD, the initial αSyn^PD^ pathology may occur in peripheral nerve terminals, whereas in other cases, the formation and deposition of αSyn^PD^ starts in the brain [105]. Accordingly, Borghammer et al. [88] proposed that PD can be possibly divided into a PNS-first and a CNS-first subtype, depending on the location of the initial αSyn^PD^ pathology. If so, the vagus nerve (and possibly also spinal-sympathetic projections) could theoretically mediate both centripetal and centrifugal spread of αSyn^PD^ pathology to and from the brain, respectively. Detection of αSyn^PD^ in peripheral tissues (e.g., the GI tract) would not then necessarily indicate starting points of αSyn^PD^ pathology [105]. 

An alternative hypothesis proposed to explain apparently centripetal spreading patterns of αSyn^PD^ pathology in PD holds that these could result from a “simultaneous and concomitant susceptibility to disease mechanisms of neurons in both the CNS and the ENS” [96]. In that case, LP and other αSyn^PD^ pathology would be governed by cell-autonomous or regionally autonomous mechanisms [28], rather than by neurally spreading αSyn^PD^ agents. In such a scenario, differences in the intrinsic cellular vulnerability for αSyn^PD^ pathology would make specific populations of neurons less fit for αSyn^PD^ clearance, or on the other hand, more susceptible to the formation of pathological αSyn aggregates [26,106]. In this context, it has been suggested that if neural trans-synaptic spread of αSyn^PD^ pathology along the gut–brain axis occurs in PD, it is gated, or modulated, by cell- or regionally autonomous mechanisms [28] and is likely to provide only one of several pathways possibly involved in the initiation and propagation of PD pathophysiology [105]. 

In any case, the unresolved challenges and open questions pointed out in this review suggest caution with regard to definitive conclusions on the neural gut–brain axis of pathological protein aggregation in PD and highlight the need to invest in further research into this important topic. Several different approaches for this are outlined in the following.

### 6.2. Possible Future Directions of Research

Animal models for PD available so far are not as realistic, powerful or robust as those for perorally acquired TSE. The significantly lower transmission efficacy of aSyn^PD^ seeds compared to prions substantially adds to this deficit. Thus, solid conclusions are more difficult to draw from studies in animals on the neural gut–brain axis in PD than in peroral prion infections. It remains to be seen whether further refined animal models or experimental designs can overcome this fundamental problem in the future. However, if animal studies will be used to further test Braak’s hypothesis on the gut–brain axis in PD, it seems advisable to focus particularly on αSyn^PD^ from PD patients. For the reasons outlined above, this seems to be more relevant and promising for the validation of the hypothesis than, for example, studies with “synthetic” fibrillar or non-fibrillar aggregates of recombinant αSyn, or with pathological αSyn species from patients with non-PD αSyn aggregation diseases (e.g., DLB or MSA). Notwithstanding this, the latter approaches have achieved important insights and may further do so in the future.

Prospectively, human organoids provide a complement or alternative to animal models in studies on the neural gut–brain axis in PD. Human cerebral organoids have been recently reported as a novel biological infection model for human prion diseases [107,108] and the generation, from human pluripotent stem cells, of 3D neuromuscular organoids forming functional neuromuscular junctions was described by Faustino Martins et al. [109]. If PD-like αSyn aggregation could be seeded and propagated in neuro-intestinal or neuro-gastric human organoids, this may open new avenues for research on PD as a whole, and the neural gut–brain axis of the disease in particular. 

Ideally, however, the trans-neuronal spreading proposed by Braak and others of αSyn^PD^ pathology via neuroanatomical pathways of the autonomic nervous system from the GI tract to the brain would be directly proven or rejected in humans. Important auxiliary evidence in this context could be obtained from epidemiological analyses of whether specific types of colectomies are associated with a reduced risk for the development of PD [105]. Similarly, it would be interesting to investigate whether colonoscopy for colorectal cancer screening, a preventive health measure in which persons with incipient PD should not be statistically overrepresented, shows an association with PD [110]. This could help to preventively address transfer risks and precautionary strategies with respect to a theoretically conceivable transmission of αSyn^PD^ seeding activity from the ENS of one person to that of another via endoscopes. At the same time, it might be also clarified whether αSyn^PD^ seeds are being transmitted in this way in real life and subsequently spread and act in recipients as would be expected according to Braak’s concept for such a transfer scenario. 

In addition to such epidemiological studies, novel lab analytical research approaches for the examination of PD patients or biological sample materials from them might shed further light on the neural gut–brain axis in PD. In vivo imaging using radiotracers and positron emission tomography (PET) provided an innovative approach to demonstrate a loss of sympathetic and parasympathetic nerve terminal function in Parkinson’s disease [111], but this examination method did not track αSyn^PD^ itself. Here, the development of specific ligands for in vivo monitoring of the distribution of pathological αSyn^PD^ species and its evolution over time, for example by longitudinal PET imaging, would provide a further advanced but technically very challenging approach [27]. 

A quantitative mapping of the temporal–spatial course of the distribution of αSyn^PD^ seeding activity in humans using highly sensitive real-time quaking induced conversion (RT-QuIC) [112,113,114,115] or protein misfolding cyclic amplification (PMCA) [116] assays could also provide important contributions to the elucidation of neuroanatomical spreading pathways of αSyn pathology in PD. αSyn^PD^ seeding activity is assumed to constitute the molecular driver underlying the propagation and spatial spread of pathological αSyn aggregation in PD. As such, it appears as a fundamental and primary indicator for the elucidation of αSyn^PD^ routing pathways covering both LP-associated as well as synaptic αSyn^PD^ seeds. In principle, analyses on the distribution and spread of αSyn^PD^ seeding activity by RT-QuIC or PMCA should be feasible with cryopreserved postmortem tissue as well as with tissue from living donors. Fixed tissue can be also analysed for αSyn^PD^ seeding activity by RT-QuIC, but possibly with reduced sensitivity, as a recent report suggests [117]. Microdissection of specific tissue areas of interest could further refine such analyses.

In perorally acquired TSE initial peripheral neuroinvasion is thought to be essentially mediated by spread of PrP^TSE^ pathology from the gut-associated lymphoid tissue (GALT) to the ENS [46,48], and the need for further investigating the involvement of the GALT in PD pathogenesis has been recently highlighted, specifically with reference to prion diseases [105]. Therefore, GALT specimens and other components of the lymphoreticular system (LRS) from PD patients should be included in tracking studies for αSyn^PD^ seeding activity. 

By pursuing the suggested approach of RT-QuIC or PMCA analyses, snapshot representations from postmortem tissues may allow for a systematic deconvolution of the temporal–spatial course of the occurrence of seeding activity, and thus αSyn^PD^ seeds, in different compartments of the gut–brain axis. Concomitantly, longitudinal studies on the development of αSyn^PD^ pathology in the ENS could be performed in patients who undergo regular gastroscopy or colonoscopy with biopsy sampling for disease monitoring or treatment. This would possibly also help to settle the controversy on reports claiming the detection of pathological αSyn^PD^ deposits in colonic and other gastro-intestinal biopsies years before the onset of PD motor symptoms [27]. That detection of αSyn^PD^ seeding activity by RT-QuIC assay in GI tissue from the stomach or colon of PD patients is feasible has been recently demonstrated in two independent studies [99,115]. Thus, using the relatively new and powerful analytical tools of RT-QuIC or PMCA for the quantitative detection of αSyn^PD^-associated seeding activity in human postmortem tissues and bioptic samples may open new perspectives on both the neural gut–brain axis of pathological protein aggregation and the ultrasensitive diagnostic detection of αSyn^PD^ seeds in PD.

## Data Availability

Not applicable.
